# *catena*-Poly[[[diacrylato-κ^4^ *O*,*O*′-neodymium(III)]-di-μ-acrylato-κ^3^ *O*,*O*′:*O*′;κ^3^ *O*:*O*,*O*′-[triaqua­neodymium(III)]-di-μ-acrylato-κ^3^ *O*,*O*′:*O*′;κ^3^ *O*:*O*,*O*′] trihydrate]

**DOI:** 10.1107/S1600536808026354

**Published:** 2008-11-08

**Authors:** Lujiang Hao, Chunhua Mu, Binbin Kong

**Affiliations:** a College of Food and Biological Engineering, Shandong Institute of Light Industry, Jinan 250353, People’s Republic of China; b Maize Research Institute, Shandong Academy of Agricultural Science, Jinan 250100, People’s Republic of China

## Abstract

The title compound, {[Nd_2_(CH_2_CHCOO)_6_(H_2_O)_3_]·3H_2_O}_*n*_, was synthesized by hydro­thermal methods. The structure contains one-dimensional coordination polymers in which two distinct Nd^III^ atoms show different coordination modes. One is coordinated by four bidentate acrylate ligands, two of which bridge Nd^III^ atoms, and by two O atoms from a further two bridging acrylate ligands. The other Nd^III^ atom is coordinated by two bidentate acrylate ligands, two O atoms from bridging acrylate ligands, and three water mol­ecules. Extensive hydrogen bonding between the coordinated and uncoordin­ated water mol­ecules and the O atoms of the acrylate ligands link the coordination polymers into a three-dimensional network.

## Related literature

For related literature, see: Church & Halvorson (1959 ▶); Chung *et al.* (1971 ▶); Okabe & Oya (2000 ▶); Okabe *et al.* (2002 ▶); Serre *et al.* (2005 ▶); Pocker & Fong (1980 ▶); Scapin *et al.* (1997 ▶).
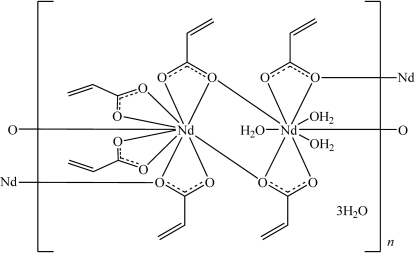


## Experimental

### 

#### Crystal data

[Nd_2_(C_3_H_3_O_2_)_6_(H_2_O)_3_]·3H_2_O
*M* *_r_* = 822.90
Monoclinic, 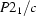

*a* = 10.2012 (10) Å
*b* = 15.242 (2) Å
*c* = 20.3073 (10) Å
β = 100.801 (2)°
*V* = 3101.7 (5) Å^3^
*Z* = 4
Mo *K*α radiation
μ = 3.38 mm^−1^
*T* = 295 (2) K
0.42 × 0.28 × 0.22 mm

#### Data collection

Bruker APEXII CCD diffractometer
Absorption correction: multi-scan (*SADABS*; Bruker, 2001 ▶) *T*_min_ = 0.300, *T*_max_ = 0.475
14403 measured reflections
5390 independent reflections
4416 reflections with *I* > 2σ(*I*)
*R*_int_ = 0.034

#### Refinement

*R*[*F* ^2^ > 2σ(*F* ^2^)] = 0.041
*wR*(*F* ^2^) = 0.109
*S* = 1.10
5390 reflections
343 parameters
72 restraints
H-atom parameters constrained
Δρ_max_ = 0.87 e Å^−3^
Δρ_min_ = −0.87 e Å^−3^

### 

Data collection: *APEX2* (Bruker, 2004 ▶); cell refinement: *SAINT-Plus* (Bruker, 2001 ▶); data reduction: *SAINT-Plus*; program(s) used to solve structure: *SHELXS97* (Sheldrick, 2008 ▶); program(s) used to refine structure: *SHELXL97* (Sheldrick, 2008 ▶); molecular graphics: *SHELXTL* (Sheldrick, 2008 ▶); software used to prepare material for publication: *SHELXTL*.

## Supplementary Material

Crystal structure: contains datablocks global, I. DOI: 10.1107/S1600536808026354/bi2299sup1.cif

Structure factors: contains datablocks I. DOI: 10.1107/S1600536808026354/bi2299Isup2.hkl

Additional supplementary materials:  crystallographic information; 3D view; checkCIF report

## Figures and Tables

**Table 1:** Hydrogen-bond geometry (Å, °)

*D*—H⋯*A*	*D*—H	H⋯*A*	*D*⋯*A*	*D*—H⋯*A*
O1—H1⋯O8	0.85	1.98	2.808 (6)	166
O1—H2⋯O16	0.85	1.92	2.751 (7)	164
O2—H3⋯O12^i^	0.85	1.91	2.723 (6)	161
O2—H4⋯O17	0.85	1.89	2.723 (7)	168
O3—H5⋯O9^i^	0.85	1.80	2.637 (7)	169
O3—H6⋯O13	0.85	2.15	2.647 (7)	117
O16—H7⋯O6^ii^	0.85	1.96	2.809 (7)	180
O16—H8⋯O17	0.85	1.92	2.772 (8)	180
O17—H9⋯O8^ii^	0.85	1.96	2.806 (7)	170
O17—H10⋯O18^iii^	0.85	2.03	2.859 (9)	163
O18—H12⋯O11^iv^	0.85	2.77	3.169 (8)	110
O18—H12⋯O14^iv^	0.85	2.29	3.139 (8)	180

Symmetry codes: (i) 
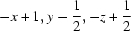
; (ii) 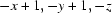; (iii) 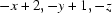; (iv) 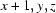.

**Fig. 1.**
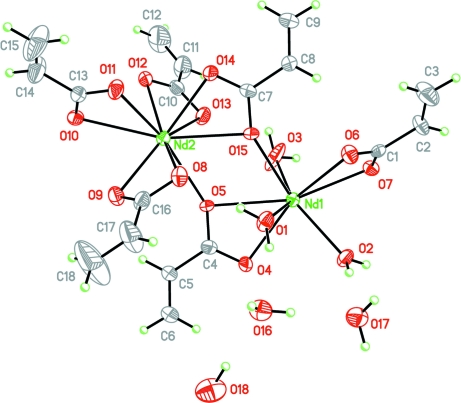
The asymmetric unit of the title compound with displacement ellipsoids drawn at 30% probability for non-H atoms.

**Fig. 2.**
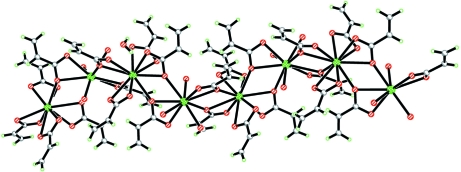
One-dimensional coordination polymers running along the [010] direction.

**Fig. 3.**
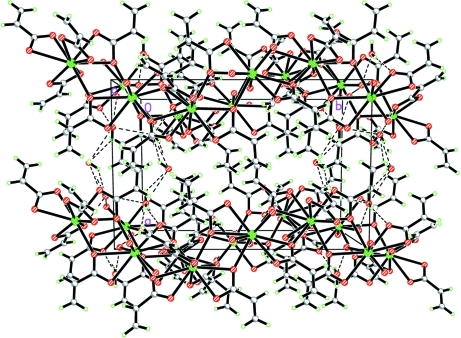
View of the packing of the title compound.

## supplementary crystallographic information

### Comment

The design of coordination compounds has received long-lasting research interest not only because of their appealing structural and topological novelty but also due to their unusual optical, electronic, magnetic and catalytic properties, and their further potential medical value derived from their antiviral and the inhibition of angiogenesis (Church & Halvorson, 1959; Chung *et al.*, 1971) and in biological systems (Okabe & Oya, 2000; Serre *et al.*, 2005; Pocker & Fong, 1980; Scapin *et al.*, 1997). To date, much of the work has been focused on coordination polymers with relatively large organic acid ligands. In this paper, we report the structure of the title Nd^III^ compound, containing acrylic acid.

### Experimental

A mixture of neodymium(III) nitrate hexahydrate (0.1 mmol), acrylic acid (0.2 mmol) and H_2_O (16 ml) in a 25 ml Teflon-lined stainless steel autoclave was kept at 473 K for three days. Violet crystals were obtained after cooling to room temperature with a yield of 6%. Elemental analysis calculated: C 26.79, H 2.98%; found: C 26.71, H 2.92%.

### Refinement

H atoms bound to C atoms were placed in calculated positions with a C—H bond distance of 0.93%A and refined as riding with *U*_iso_(H) = 1.2*U*_eq_(C). The H atoms of the water molecules were placed so as to form a reasonable hydrogen-bond network, with O—H = 0.85 Å, and refined as riding with *U*_iso_(H) = 1.5*U*_eq_(O). The anisotropic displacement parameters of the two terminal C atoms of each acrylate ligand were restrained to approximate isotropic behaviour.

### Crystal data

**Table:**

[Nd_2_(C_3_H_3_O_2_)_6_(H_2_O)_3_]·3H_2_O	*F*_000_ = 1608
*M**_r_* = 822.90	*D*_x_ = 1.749 Mg m^−^^3^
Monoclinic, *P*2_1_/*c*	Mo *K*α radiation λ = 0.71073 Å
Hall symbol: -P 2ybc	Cell parameters from 5390 reflections
*a* = 10.2012 (10) Å	θ = 1.7–25.1º
*b* = 15.242 (2) Å	µ = 3.38 mm^−^^1^
*c* = 20.3073 (10) Å	*T* = 295 (2) K
β = 100.801 (2)º	Block, violet
*V* = 3101.7 (5) Å^3^	0.42 × 0.28 × 0.22 mm
*Z* = 4	

### Data collection

**Table:**

Bruker APEXII CCD diffractometer	5390 independent reflections
Radiation source: fine-focus sealed tube	4416 reflections with *I* > 2σ(*I*)
Monochromator: graphite	*R*_int_ = 0.034
*T* = 295(2) K	θ_max_ = 25.1º
φ and ω scans	θ_min_ = 1.7º
Absorption correction: multi-scan(SADABS; Bruker, 2001)	*h* = −12→10
*T*_min_ = 0.300, *T*_max_ = 0.475	*k* = −18→18
14403 measured reflections	*l* = −21→24

### Refinement

**Table:**

Refinement on *F*^2^	Secondary atom site location: difference Fourier map
Least-squares matrix: full	Hydrogen site location: inferred from neighbouring sites
*R*[*F*^2^ > 2σ(*F*^2^)] = 0.041	H-atom parameters constrained
*wR*(*F*^2^) = 0.109	*w* = 1/[σ^2^(*F*_o_^2^) + (0.0473*P*)^2^ + 8.3226*P*] where *P* = (*F*_o_^2^ + 2*F*_c_^2^)/3
*S* = 1.10	(Δ/σ)_max_ = 0.001
5390 reflections	Δρ_max_ = 0.87 e Å^−^^3^
343 parameters	Δρ_min_ = −0.87 e Å^−^^3^
72 restraints	Extinction correction: none
Primary atom site location: structure-invariant direct methods	

### Special details

**Table:**

Geometry. All esds (except the esd in the dihedral angle between two l.s. planes) are estimated using the full covariance matrix. The cell esds are taken into account individually in the estimation of esds in distances, angles and torsion angles; correlations between esds in cell parameters are only used when they are defined by crystal symmetry. An approximate (isotropic) treatment of cell esds is used for estimating esds involving l.s. planes.
Refinement. Refinement of *F*^2^ against ALL reflections. The weighted *R*-factor *wR* and goodness of fit *S* are based on *F*^2^, conventional *R*-factors *R* are based on *F*, with *F* set to zero for negative *F*^2^. The threshold expression of *F*^2^ > σ(*F*^2^) is used only for calculating *R*-factors(gt) *etc*. and is not relevant to the choice of reflections for refinement. *R*-factors based on *F*^2^ are statistically about twice as large as those based on *F*, and *R*- factors based on ALL data will be even larger.

### Fractional atomic coordinates and isotropic or equivalent isotropic displacement parameters (Å^2^)

**Table:**

	*x*	*y*	*z*	*U*_iso_*/*U*_eq_	
Nd1	0.56385 (3)	0.45807 (2)	0.191128 (18)	0.03537 (12)	
Nd2	0.35529 (3)	0.70432 (2)	0.209240 (18)	0.03519 (12)	
C1	0.4158 (6)	0.2907 (4)	0.1465 (3)	0.0340 (14)	
C2	0.3402 (8)	0.2089 (5)	0.1262 (4)	0.0483 (18)	
H2A	0.3751	0.1561	0.1448	0.058*	
C3	0.2300 (10)	0.2073 (7)	0.0846 (5)	0.080 (3)	
H3A	0.1931	0.2592	0.0654	0.096*	
H3B	0.1864	0.1542	0.0735	0.096*	
C4	0.7001 (6)	0.6303 (4)	0.2170 (3)	0.0395 (15)	
C5	0.7686 (7)	0.7146 (5)	0.2180 (4)	0.0500 (18)	
H5A	0.7206	0.7657	0.2217	0.060*	
C6	0.8912 (9)	0.7210 (6)	0.2139 (5)	0.076 (3)	
H6A	0.9410	0.6707	0.2102	0.092*	
H6B	0.9311	0.7760	0.2147	0.092*	
C7	0.2188 (7)	0.5346 (4)	0.1710 (4)	0.0417 (16)	
C8	0.1496 (8)	0.4493 (5)	0.1711 (5)	0.067 (2)	
H8A	0.1979	0.3980	0.1687	0.080*	
C9	0.0265 (10)	0.4431 (7)	0.1744 (6)	0.093 (3)	
H9A	−0.0237	0.4936	0.1768	0.111*	
H9B	−0.0133	0.3882	0.1743	0.111*	
C10	0.2684 (7)	0.6554 (5)	0.3355 (4)	0.0459 (17)	
C11	0.2344 (11)	0.6327 (6)	0.4014 (5)	0.080 (3)	
H11A	0.2777	0.5852	0.4247	0.097*	
C12	0.1484 (14)	0.6749 (9)	0.4281 (7)	0.115 (4)	
H12A	0.1036	0.7226	0.4059	0.138*	
H12B	0.1311	0.6576	0.4695	0.138*	
C13	0.1885 (7)	0.8637 (4)	0.1723 (4)	0.0456 (17)	
C14	0.0995 (10)	0.9380 (6)	0.1499 (5)	0.078 (3)	
H14A	0.1112	0.9901	0.1741	0.094*	
C15	0.0045 (12)	0.9334 (8)	0.0971 (6)	0.114 (4)	
H15A	−0.0087	0.8818	0.0723	0.137*	
H15B	−0.0500	0.9817	0.0843	0.137*	
C16	0.4621 (8)	0.7649 (5)	0.0902 (4)	0.0476 (17)	
C17	0.5220 (12)	0.7930 (7)	0.0339 (5)	0.091 (3)	
H17A	0.5154	0.7577	−0.0039	0.110*	
C18	0.5862 (17)	0.8694 (12)	0.0366 (9)	0.168 (7)	
H18A	0.5929	0.9048	0.0744	0.201*	
H18B	0.6244	0.8873	0.0007	0.201*	
O1	0.5484 (5)	0.5364 (3)	0.0820 (2)	0.0486 (12)	
H1	0.5011	0.5826	0.0761	0.073*	
H2	0.6054	0.5372	0.0564	0.073*	
O2	0.7108 (5)	0.3755 (3)	0.1297 (2)	0.0473 (12)	
H3	0.7501	0.3300	0.1480	0.071*	
H4	0.7247	0.3802	0.0899	0.071*	
O3	0.4934 (8)	0.4659 (4)	0.3008 (3)	0.093 (3)	
H5	0.5148	0.4174	0.3206	0.139*	
H6	0.4108	0.4757	0.2991	0.139*	
O4	0.7608 (5)	0.5595 (3)	0.2143 (3)	0.0539 (14)	
O5	0.5747 (4)	0.6278 (3)	0.2182 (2)	0.0341 (9)	
O6	0.3974 (5)	0.3575 (3)	0.1107 (2)	0.0450 (11)	
O7	0.4972 (4)	0.2917 (3)	0.2019 (2)	0.0364 (10)	
O8	0.4008 (5)	0.6912 (3)	0.0861 (2)	0.0473 (12)	
O9	0.4736 (5)	0.8099 (3)	0.1423 (2)	0.0476 (12)	
O10	0.2794 (4)	0.8729 (3)	0.2236 (2)	0.0392 (10)	
O11	0.1697 (5)	0.7925 (3)	0.1422 (3)	0.0525 (13)	
O12	0.2189 (5)	0.7220 (3)	0.3044 (3)	0.0457 (12)	
O13	0.3521 (5)	0.6084 (3)	0.3138 (3)	0.0508 (12)	
O14	0.1589 (4)	0.6050 (3)	0.1722 (3)	0.0539 (14)	
O15	0.3457 (4)	0.5347 (3)	0.1730 (3)	0.0438 (12)	
O16	0.7445 (5)	0.5710 (4)	0.0093 (3)	0.0594 (14)	
H7	0.7020	0.5927	−0.0271	0.089*	
H8	0.7596	0.5161	0.0095	0.089*	
O17	0.7927 (5)	0.3919 (4)	0.0102 (3)	0.0590 (14)	
H9	0.7296	0.3650	−0.0151	0.088*	
H10	0.8651	0.3843	−0.0040	0.088*	
O18	0.9798 (6)	0.6714 (5)	0.0387 (3)	0.091 (2)	
H11	0.9085	0.6413	0.0298	0.137*	
H12	1.0283	0.6535	0.0749	0.137*	

### Atomic displacement parameters (Å^2^)

**Table:**

	*U*^11^	*U*^22^	*U*^33^	*U*^12^	*U*^13^	*U*^23^
Nd1	0.0335 (2)	0.0297 (2)	0.0442 (2)	0.00266 (13)	0.01071 (16)	0.00193 (14)
Nd2	0.0305 (2)	0.0306 (2)	0.0441 (2)	0.00072 (13)	0.00585 (15)	−0.00324 (14)
C1	0.032 (3)	0.037 (3)	0.038 (3)	0.003 (3)	0.019 (3)	−0.004 (3)
C2	0.053 (4)	0.042 (4)	0.046 (4)	−0.015 (3)	−0.002 (3)	−0.003 (3)
C3	0.074 (6)	0.074 (5)	0.084 (6)	−0.021 (4)	−0.005 (5)	0.000 (5)
C4	0.035 (4)	0.044 (4)	0.041 (4)	−0.003 (3)	0.011 (3)	0.000 (3)
C5	0.043 (4)	0.042 (4)	0.069 (5)	−0.005 (3)	0.019 (3)	−0.003 (3)
C6	0.061 (5)	0.058 (5)	0.117 (7)	−0.014 (4)	0.033 (5)	−0.011 (5)
C7	0.032 (4)	0.043 (4)	0.052 (4)	−0.006 (3)	0.012 (3)	−0.001 (3)
C8	0.044 (4)	0.046 (4)	0.116 (7)	−0.002 (3)	0.027 (4)	−0.009 (4)
C9	0.059 (5)	0.074 (6)	0.151 (8)	−0.012 (5)	0.035 (5)	−0.007 (6)
C10	0.049 (4)	0.039 (4)	0.056 (4)	−0.012 (3)	0.025 (4)	−0.009 (3)
C11	0.100 (6)	0.065 (5)	0.089 (6)	0.002 (5)	0.052 (5)	0.006 (5)
C12	0.134 (8)	0.110 (7)	0.120 (8)	−0.011 (7)	0.069 (7)	0.009 (6)
C13	0.038 (4)	0.036 (4)	0.062 (5)	0.002 (3)	0.007 (3)	0.005 (3)
C14	0.080 (6)	0.059 (5)	0.085 (6)	0.018 (4)	−0.013 (5)	−0.006 (4)
C15	0.108 (7)	0.099 (7)	0.117 (7)	0.039 (6)	−0.027 (6)	−0.004 (6)
C16	0.059 (5)	0.040 (4)	0.048 (4)	0.009 (3)	0.020 (4)	0.000 (3)
C17	0.133 (8)	0.079 (6)	0.075 (6)	−0.033 (6)	0.055 (6)	−0.009 (5)
C18	0.205 (11)	0.167 (10)	0.153 (10)	−0.040 (8)	0.089 (8)	−0.012 (8)
O1	0.068 (3)	0.039 (3)	0.045 (3)	0.014 (2)	0.025 (2)	0.012 (2)
O2	0.055 (3)	0.039 (3)	0.055 (3)	0.014 (2)	0.028 (2)	0.007 (2)
O3	0.154 (7)	0.070 (4)	0.076 (4)	0.075 (4)	0.074 (4)	0.039 (3)
O4	0.032 (3)	0.029 (2)	0.100 (4)	0.001 (2)	0.011 (3)	0.000 (3)
O5	0.021 (2)	0.034 (2)	0.048 (3)	0.0008 (17)	0.0099 (18)	−0.003 (2)
O6	0.049 (3)	0.039 (3)	0.043 (3)	−0.001 (2)	−0.001 (2)	0.004 (2)
O7	0.032 (2)	0.039 (2)	0.035 (2)	−0.0001 (18)	−0.0015 (19)	0.0082 (19)
O8	0.056 (3)	0.038 (3)	0.047 (3)	0.001 (2)	0.007 (2)	−0.004 (2)
O9	0.064 (3)	0.037 (3)	0.048 (3)	−0.008 (2)	0.023 (2)	−0.005 (2)
O10	0.028 (2)	0.042 (3)	0.043 (3)	−0.0012 (19)	−0.004 (2)	−0.005 (2)
O11	0.044 (3)	0.038 (3)	0.066 (3)	0.009 (2)	−0.014 (2)	−0.005 (2)
O12	0.045 (3)	0.032 (2)	0.065 (3)	−0.003 (2)	0.023 (2)	−0.002 (2)
O13	0.060 (3)	0.038 (3)	0.060 (3)	0.010 (2)	0.026 (3)	0.002 (2)
O14	0.030 (2)	0.037 (3)	0.091 (4)	0.000 (2)	0.002 (2)	−0.016 (3)
O15	0.022 (2)	0.039 (3)	0.070 (3)	−0.0021 (18)	0.011 (2)	−0.007 (2)
O16	0.056 (3)	0.068 (4)	0.052 (3)	0.000 (3)	0.006 (3)	0.013 (3)
O17	0.056 (3)	0.067 (4)	0.054 (3)	−0.007 (3)	0.011 (3)	−0.003 (3)
O18	0.057 (4)	0.135 (6)	0.075 (4)	0.002 (4)	−0.008 (3)	−0.010 (4)

### Geometric parameters (Å, °)

**Table:**

Nd1—O2	2.467 (4)	C8—C9	1.273 (12)
Nd1—O3	2.468 (6)	C8—H8A	0.930
Nd1—O15	2.479 (4)	C9—H9A	0.930
Nd1—O10^i^	2.490 (4)	C9—H9B	0.930
Nd1—O1	2.496 (4)	C10—O12	1.250 (9)
Nd1—O4	2.508 (5)	C10—O13	1.256 (8)
Nd1—O6	2.619 (5)	C10—C11	1.485 (12)
Nd1—O5	2.643 (4)	C11—C12	1.286 (15)
Nd1—O7	2.645 (4)	C11—H11A	0.930
Nd1—C4	2.971 (7)	C12—H12A	0.930
Nd1—C1	3.016 (6)	C12—H12B	0.930
Nd2—O5	2.500 (4)	C13—O11	1.243 (8)
Nd2—O7^ii^	2.505 (4)	C13—O10	1.266 (8)
Nd2—O11	2.506 (4)	C13—C14	1.470 (11)
Nd2—O14	2.511 (4)	C14—C15	1.305 (14)
Nd2—O9	2.552 (5)	C14—H14A	0.930
Nd2—O13	2.583 (5)	C15—H15A	0.930
Nd2—O12	2.598 (5)	C15—H15B	0.930
Nd2—O8	2.636 (5)	C16—O9	1.247 (8)
Nd2—O15	2.685 (4)	C16—O8	1.281 (9)
Nd2—O10	2.716 (4)	C16—C17	1.459 (12)
Nd2—C10	2.961 (7)	C17—C18	1.331 (17)
Nd2—C7	2.972 (7)	C17—H17A	0.930
C1—O6	1.246 (7)	C18—H18A	0.930
C1—O7	1.267 (7)	C18—H18B	0.930
C1—C2	1.483 (9)	O1—H1	0.850
C2—C3	1.274 (11)	O1—H2	0.850
C2—H2A	0.930	O2—H3	0.850
C3—H3A	0.930	O2—H4	0.850
C3—H3B	0.930	O3—H5	0.850
C4—O4	1.251 (8)	O3—H6	0.850
C4—O5	1.285 (7)	O7—Nd2^i^	2.505 (4)
C4—C5	1.461 (9)	O10—Nd1^ii^	2.490 (4)
C5—C6	1.273 (11)	O16—H7	0.850
C5—H5A	0.930	O16—H8	0.850
C6—H6A	0.930	O17—H9	0.850
C6—H6B	0.930	O17—H10	0.850
C7—O14	1.238 (8)	O18—H11	0.850
C7—O15	1.288 (8)	O18—H12	0.850
C7—C8	1.480 (10)		
			
O2—Nd1—O3	141.98 (17)	O14—Nd2—C7	24.29 (16)
O2—Nd1—O15	141.19 (16)	O9—Nd2—C7	130.91 (17)
O3—Nd1—O15	72.91 (19)	O13—Nd2—C7	69.20 (18)
O2—Nd1—O10^i^	73.36 (15)	O12—Nd2—C7	89.70 (17)
O3—Nd1—O10^i^	69.83 (17)	O8—Nd2—C7	80.95 (17)
O15—Nd1—O10^i^	142.21 (16)	O15—Nd2—C7	25.68 (16)
O2—Nd1—O1	74.85 (15)	O10—Nd2—C7	136.31 (16)
O3—Nd1—O1	142.90 (16)	C10—Nd2—C7	78.80 (19)
O15—Nd1—O1	74.78 (17)	O6—C1—O7	120.7 (6)
O10^i^—Nd1—O1	142.37 (16)	O6—C1—C2	120.8 (6)
O2—Nd1—O4	82.42 (17)	O7—C1—C2	118.4 (6)
O3—Nd1—O4	99.1 (2)	O6—C1—Nd1	59.7 (3)
O15—Nd1—O4	113.74 (14)	O7—C1—Nd1	61.0 (3)
O10^i^—Nd1—O4	78.65 (15)	C2—C1—Nd1	178.1 (5)
O1—Nd1—O4	77.71 (18)	C3—C2—C1	123.2 (8)
O2—Nd1—O6	76.75 (16)	C3—C2—H2A	118.4
O3—Nd1—O6	109.1 (2)	C1—C2—H2A	118.4
O15—Nd1—O6	73.98 (15)	C2—C3—H3A	120.0
O10^i^—Nd1—O6	112.68 (14)	C2—C3—H3B	120.0
O1—Nd1—O6	78.48 (16)	H3A—C3—H3B	120.0
O4—Nd1—O6	151.69 (18)	O4—C4—O5	118.6 (6)
O2—Nd1—O5	126.78 (14)	O4—C4—C5	121.4 (6)
O3—Nd1—O5	76.88 (17)	O5—C4—C5	120.1 (6)
O15—Nd1—O5	64.60 (13)	O4—C4—Nd1	56.5 (3)
O10^i^—Nd1—O5	111.66 (14)	O5—C4—Nd1	62.8 (3)
O1—Nd1—O5	73.12 (14)	C5—C4—Nd1	170.5 (5)
O4—Nd1—O5	49.98 (13)	C6—C5—C4	122.7 (8)
O6—Nd1—O5	134.37 (13)	C6—C5—H5A	118.7
O2—Nd1—O7	75.09 (15)	C4—C5—H5A	118.7
O3—Nd1—O7	81.30 (18)	C5—C6—H6A	120.0
O15—Nd1—O7	103.09 (14)	C5—C6—H6B	120.0
O10^i^—Nd1—O7	65.44 (13)	H6A—C6—H6B	120.0
O1—Nd1—O7	123.89 (15)	O14—C7—O15	119.7 (6)
O4—Nd1—O7	141.67 (14)	O14—C7—C8	121.6 (6)
O6—Nd1—O7	49.02 (13)	O15—C7—C8	118.6 (6)
O5—Nd1—O7	157.32 (14)	O14—C7—Nd2	56.5 (3)
O2—Nd1—C4	103.41 (17)	O15—C7—Nd2	64.6 (3)
O3—Nd1—C4	90.2 (2)	C8—C7—Nd2	164.7 (6)
O15—Nd1—C4	89.26 (16)	C9—C8—C7	122.7 (8)
O10^i^—Nd1—C4	96.84 (16)	C9—C8—H8A	118.7
O1—Nd1—C4	71.59 (17)	C7—C8—H8A	118.7
O4—Nd1—C4	24.58 (16)	C8—C9—H9A	120.0
O6—Nd1—C4	148.70 (16)	C8—C9—H9B	120.0
O5—Nd1—C4	25.61 (15)	H9A—C9—H9B	120.0
O7—Nd1—C4	162.07 (16)	O12—C10—O13	121.5 (7)
O2—Nd1—C1	74.59 (16)	O12—C10—C11	120.3 (7)
O3—Nd1—C1	95.6 (2)	O13—C10—C11	118.2 (7)
O15—Nd1—C1	88.21 (15)	O12—C10—Nd2	61.1 (4)
O10^i^—Nd1—C1	89.33 (16)	O13—C10—Nd2	60.4 (4)
O1—Nd1—C1	101.13 (16)	C11—C10—Nd2	175.9 (6)
O4—Nd1—C1	156.34 (16)	C12—C11—C10	123.8 (11)
O6—Nd1—C1	24.25 (15)	C12—C11—H11A	118.1
O5—Nd1—C1	152.81 (14)	C10—C11—H11A	118.1
O7—Nd1—C1	24.78 (15)	C11—C12—H12A	120.0
C4—Nd1—C1	172.70 (18)	C11—C12—H12B	120.0
O5—Nd2—O7^ii^	77.69 (14)	H12A—C12—H12B	120.0
O5—Nd2—O11	149.84 (17)	O11—C13—O10	121.6 (6)
O7^ii^—Nd2—O11	113.40 (15)	O11—C13—C14	119.4 (7)
O5—Nd2—O14	113.20 (14)	O10—C13—C14	118.9 (7)
O7^ii^—Nd2—O14	150.92 (17)	O11—C13—Nd2	56.0 (3)
O11—Nd2—O14	71.41 (16)	O10—C13—Nd2	65.7 (3)
O5—Nd2—O9	80.01 (15)	C14—C13—Nd2	175.3 (6)
O7^ii^—Nd2—O9	76.61 (15)	C15—C14—C13	122.3 (10)
O11—Nd2—O9	75.91 (17)	C15—C14—H14A	118.9
O14—Nd2—O9	130.62 (18)	C13—C14—H14A	118.9
O5—Nd2—O13	79.93 (15)	C14—C15—H15A	120.0
O7^ii^—Nd2—O13	78.88 (16)	C14—C15—H15B	120.0
O11—Nd2—O13	128.74 (18)	H15A—C15—H15B	120.0
O14—Nd2—O13	76.87 (18)	O9—C16—O8	120.4 (7)
O9—Nd2—O13	151.06 (17)	O9—C16—C17	121.0 (7)
O5—Nd2—O12	126.24 (15)	O8—C16—C17	118.6 (7)
O7^ii^—Nd2—O12	75.06 (15)	O9—C16—Nd2	58.2 (4)
O11—Nd2—O12	83.88 (17)	O8—C16—Nd2	62.2 (4)
O14—Nd2—O12	77.10 (16)	C17—C16—Nd2	176.7 (7)
O9—Nd2—O12	134.73 (14)	C18—C17—C16	120.1 (11)
O13—Nd2—O12	49.93 (15)	C18—C17—H17A	120.0
O5—Nd2—O8	73.69 (15)	C16—C17—H17A	120.0
O7^ii^—Nd2—O8	122.36 (15)	C17—C18—H18A	120.0
O11—Nd2—O8	76.95 (17)	C17—C18—H18B	120.0
O14—Nd2—O8	86.70 (17)	H18A—C18—H18B	120.0
O9—Nd2—O8	50.00 (15)	Nd1—O1—H1	117.5
O13—Nd2—O8	140.23 (14)	Nd1—O1—H2	127.9
O12—Nd2—O8	158.09 (16)	H1—O1—H2	109.7
O5—Nd2—O15	63.70 (13)	Nd1—O2—H3	118.6
O7^ii^—Nd2—O15	134.05 (14)	Nd1—O2—H4	131.5
O11—Nd2—O15	112.49 (15)	H3—O2—H4	109.5
O14—Nd2—O15	49.58 (14)	Nd1—O3—H5	107.0
O9—Nd2—O15	117.26 (15)	Nd1—O3—H6	115.2
O13—Nd2—O15	71.02 (16)	H5—O3—H6	109.7
O12—Nd2—O15	107.85 (14)	C4—O4—Nd1	98.9 (4)
O8—Nd2—O15	70.77 (15)	C4—O5—Nd2	150.0 (4)
O5—Nd2—O10	134.66 (13)	C4—O5—Nd1	91.6 (4)
O7^ii^—Nd2—O10	64.17 (13)	Nd2—O5—Nd1	116.07 (15)
O11—Nd2—O10	49.41 (14)	C1—O6—Nd1	96.0 (4)
O14—Nd2—O10	112.03 (14)	C1—O7—Nd2^i^	147.0 (4)
O9—Nd2—O10	68.48 (14)	C1—O7—Nd1	94.2 (4)
O13—Nd2—O10	113.55 (14)	Nd2^i^—O7—Nd1	116.12 (15)
O12—Nd2—O10	67.59 (14)	C16—O8—Nd2	92.4 (4)
O8—Nd2—O10	106.17 (14)	C16—O9—Nd2	97.2 (4)
O15—Nd2—O10	160.80 (13)	C13—O10—Nd1^ii^	155.0 (4)
O5—Nd2—C10	103.23 (18)	C13—O10—Nd2	89.2 (4)
O7^ii^—Nd2—C10	75.35 (17)	Nd1^ii^—O10—Nd2	114.15 (15)
O11—Nd2—C10	106.7 (2)	C13—O11—Nd2	99.7 (4)
O14—Nd2—C10	75.88 (19)	C10—O12—Nd2	94.0 (4)
O9—Nd2—C10	150.29 (17)	C10—O13—Nd2	94.6 (4)
O13—Nd2—C10	25.02 (17)	C7—O14—Nd2	99.2 (4)
O12—Nd2—C10	24.91 (17)	C7—O15—Nd1	150.9 (4)
O8—Nd2—C10	159.58 (16)	C7—O15—Nd2	89.7 (4)
O15—Nd2—C10	89.62 (17)	Nd1—O15—Nd2	115.31 (16)
O10—Nd2—C10	90.43 (17)	H7—O16—H8	116.9
O5—Nd2—C7	88.98 (16)	H9—O17—H10	109.7
O7^ii^—Nd2—C7	147.14 (17)	H11—O18—H12	110.4
O11—Nd2—C7	93.18 (17)		

Symmetry codes: (i) −*x*+1, *y*−1/2, −*z*+1/2; (ii) −*x*+1, *y*+1/2, −*z*+1/2.

### Hydrogen-bond geometry (Å, °)

**Table:**

*D*—H···*A*	*D*—H	H···*A*	*D*···*A*	*D*—H···*A*
O1—H1···O8	0.85	1.98	2.808 (6)	166
O1—H2···O16	0.85	1.92	2.751 (7)	164
O2—H3···O12^i^	0.85	1.91	2.723 (6)	161
O2—H4···O17	0.85	1.89	2.723 (7)	168
O3—H5···O9^i^	0.85	1.80	2.637 (7)	169
O3—H6···O13	0.85	2.15	2.647 (7)	117
O16—H7···O6^iii^	0.85	1.96	2.809 (7)	180
O16—H8···O17	0.85	1.92	2.772 (8)	180
O17—H9···O8^iii^	0.85	1.96	2.806 (7)	170
O17—H10···O18^iv^	0.85	2.03	2.859 (9)	163
O18—H12···O11^v^	0.85	2.77	3.169 (8)	110
O18—H12···O14^v^	0.85	2.29	3.139 (8)	180

Symmetry codes: (i) −*x*+1, *y*−1/2, −*z*+1/2; (iii) −*x*+1, −*y*+1, −*z*; (iv) −*x*+2, −*y*+1, −*z*; (v) *x*+1, *y*, *z*.
